# Supplementary material for: A global comparison of the nutritive values of forage plants grown in contrasting environments
Source: J Plant Res. 2018 Mar 17;131(4):641–54. doi: 10.1007/s10265-018-1024-y (PMC6015622; doi:10.1007/s10265-018-1024-y)
Supplement: Supplementary file 1 — Supplementary material 1 (PDF 349 KB) [file 10265_2018_1024_MOESM1_ESM.pdf]

Electric supplementary materials

Title:

A global comparison of the nutritive values of forage plants grown in contrasting environments

Authors:

Mark A. Lee

Journal:

Journal of Plant Research

Corresponding author:

Mark A. Lee

Natural Capital and Plant Health

Royal Botanic Gardens Kew

TW9 3AB

UK

+44 (0)20 8332 5000

m.lee@kew.org

Content:

Tables S1–S2

Table S1 Articles included in the database including author, year of publication, country, site name, site latitude (lat) and longitude (long), mean annual rainfall (MAR), mean annual temperature (MAT), altitude (alt) and bioclimatic zone. Zones were allocated as per the Koppen-Geiger classification. Note that some articles refer to more than one site.

Reference	Country	Lat	Long	MAR	MAT	Alt	Zone
Ajayi et al. 2008	Nigeria	7.25	3.75	1349.1	26.2	161	Equatorial
Akgun et al. 2008	Turkey	39.92	41.27	37.9	4.4	1850	Tundra
Al-Masri 2013	Syria	35.37	39.77	148.4	20.6	203	Arid
Albayrak and Türk 2013	Turkey	37.75	30.55	498.1	12.8	1035	Temperate
Al-Ghumaiz and Motawei 2011	Saudi Arabia	26.31	43.77	160.6	24.7	652	Arid
Amole et al. 2015	Nigeria	7.00	3.50	1037.0	-	155	Equatorial
Andrzejewska et al. 2016	Poland	54.08	18.82	600.5	8.3	4	Temperate
Andrzejewska et al. 2016	Poland	53.20	17.85	566.6	-	95	Temperate
Annese et al. 2006	Italy	41.10	15.85	450.0	-	180	Temperate
Balseca et al. 2015	Ecuador	-0.23	-79.25	2700.0	23.5	425	Equatorial
Beecher et al. 2015	Ireland	52.16	-8.26	1040.0	10.0	70	Temperate
Bélanger and Mcqueen 1997	Canada	45.92	-66.60	1065.0	5.6	26	Tundra
Berhane et al. 2006	Ethiopia	13.46	39.02	-	-	1375	Temperate
Bryant et al. 2012	New Zealand	-43.63	172.47	581.2	11.5	22	Temperate
Callow et al. 2003	Australia	-27.77	152.67	815.0	19.9	40	Temperate
Catanese et al. 2009	Argentina	-37.18	-62.13	602.7	15.9	181	Temperate
Cherney and Cherney 1997	United States	41.11	-73.81	1327.0	11.5	100	Temperate
Cherney and Cherney 1997	United States	42.44	-76.50	963.9	8.4	120	Tundra
Conaghan et al. 2008	Ireland	53.50	-6.67	877.3	6.3	83	Temperate
Čop et al. 2009	Slovenia	46.05	14.47	914.8	10.8	300	Temperate
Distel et al. 2005	Argentina	-38.45	-63.75	400.0	15.0	95	Temperate
Dong et al. 2003	China	37.67	103.53	385.7	-1.0	3000	Arid
dos Santos et al. 2003	Brazil	-8.01	-34.95	2310.3	25.7	23	Equatorial
Drobná and Jančovič 2006	Slovakia	48.59	17.83	595.0	9.2	163	Temperate
Ducati et al. 2015	Brazil	-24.56	-54.07	450.0	22.5	419	Temperate
Elgersma et al. 2014	Denmark	56.48	9.57	-	-	51	Temperate
Fraser et al. 2004	UK	52.42	-4.08	1174.0	10.0	230	Temperate
Gierus et al. 2012	Germany	54.45	9.95	811.0	10.2	20	Temperate
Golaszewska et al	Poland	53.59	19.86	626.2	7.4	151	Temperate
Griggs et al. 2007	United States	41.77	-111.82	509.6	9.1	1406	Tundra
Haferkamp and Grings 2002	United States	46.37	-105.08	498.3	8.2	719	Arid
Hirata et al. 2008	Japan	31.98	131.47	2378.0	17.3	11	Temperate
Homolka et al. 2012	Czech Republic	50.04	14.63	626.0	8.9	240	Temperate
Kambashi et al. 2014	DRC	5.42	14.82	-	-	926	Equatorial
Karadag and Buyukburc 2004	Turkey	40.22	36.02	471.9	11.8	623	Temperate
Keating and O'Kiely 2000	Ireland	53.50	-6.67	877.3	6.3	92	Temperate
King et al. 2012	Ireland	53.50	-6.67	877.3	6.3	83	Temperate
Kobayashi et al. 2008	Japan	35.17	132.50	1603.9	15.9	53	Temperate

Reference	Country	Lat	Long	MAR	MAT	Alt	Zone
Lee et al. 2001	UK	52.37	-4.08	1174.0	10.0	100	Temperate
Maasdorp et al. 1999	Zimbabwe	-17.50	31.03	815.0	18.3	1480	Temperate
McCartney et al. 2008	Canada	52.82	-104.60	439.0	0.7	483	Tundra
McCartney et al. 2008	Canada	52.47	-113.73	466.0	2.4	855	Tundra
Mceniry et al. 2014	Ireland	53.50	-6.67	877.3	6.3	83	Temperate
Miguel et al. 2012	Brazil	-27.78	-50.31	1578.1	20.7	920	Temperate
Mtengeti et al. 2008	Tanzania	-5.12	37.63	900.0	-	400	Equatorial
Mupangwa et al. 1997	Zimbabwe	-17.65	31.61	848.0	29.0	1254	Temperate
Nashiki et al. 2005	Japan	39.73	141.13	1180.0	9.3	110	Tundra
Ramirez 2007	Mexico	25.72	-100.03	500.0	22.0	393	Arid
Ramirez et al. 2009	Mexico	25.41	-99.78	360.0	22.0	272	Arid
Safari et al. 2011	Tanzania	-4.42	35.20	646.0	-	1791	Equatorial
Sahin et al. 2012	Turkey	39.91	41.27	409.4	5.7	1905	Tundra
Santos et al. 2016	Brazil	0.00	0.00	-	-	-	Arid
Skladanka et al. 2010	Czech Republic	49.52	15.97	617.0	6.9	560	Temperate
Smit et al. 2005	Netherlands	51.97	5.67	771.4	9.3	7	Temperate
Stout et al. 1997	Canada	50.70	-120.40	257.0	9.5	349	Tundra
Suleiman et al. 1999	Canada	53.76	-113.34	455.8	3.0	674	Tundra
Surmen et al. 2013	Turkey	41.24	36.51	709.3	14.6	4	Temperate
Taffarel et al. 2016	Brazil	-24.56	-54.07	1720.0	22.5	420	Temperate
Tas et al. 2005	Netherlands	51.97	5.67	771.4	9.3	7	Temperate
Tefera et al. 2009	Swaziland	-26.89	31.94	-	-	116	Temperate
Tefera et al. 2009	Swaziland	-26.22	31.92	-	-	259	Temperate
Ulyatt et al. 2002	New Zealand	-35.30	173.90	-	-	83	Temperate
Weller and Cooper 2001	UK	52.27	-4.08	1823.8	10.0	257	Temperate
Yayneshet et al. 2009	Ethiopia	13.70	40.25	519.0	21.6	143	Arid
Yayneshet et al. 2009	Ethiopia	13.72	39.53	500.0	19.4	2000	Arid
Zemenchik et al. 2002	United States	43.30	-89.35	-	-	317	Tundra
Zemenchik et al. 2002	United States	42.83	-90.78	-	-	313	Tundra
Zhao et al. 2012	Mongolia	42.26	118.93	380.0	4.5	900	Arid

Table S2 Mean dry matter (DM), acid detergent lignin (ADL), acid detergent fibre (ADF), mineral ash (Ash), crude protein (CP), neutral detergent fibre (NDF), dry matter digestibility (DMD) and organic matter digestibility (OMD). All values are states as percent by dry matter (% DM). Hybrids are denoted by an “x”.

Species	ADF	ADL	Ash	CP	DM	NDF	DMD	OMD
Acacia boliviana	30	-	-	16	91	59	-	57
Acacia etbaica	25	11	7	17	91	40	-	-
Acacia seyal	22	7	8	17	89	38	-	-
Aeschynomene histrix	31	8	8	21	40	62	-	-
Agropyron cristatum	31	-	-	17	-	-	64	8
Albizia amara	34	16	7	21	91	58	-	-
Alopecurus pratensis	33	-	-	15	-	58	-	-
Andropogon gayanus	47	16	10	7	93	64	-	-
Aristida adscensionis	50	9	-	5	-	79	41	-
Aristida longiseta	-	-	-	-	-	87	-	-
Aristida sciurus	38	-	-	5	90	61	-	-
Arrhenatherum elatius	-	-	-	8	-	61	-	74
Artemisia herba-alba	47	-	10	10	-	57	-	-
Astragalus spinosus	53	-	8	10	-	64	-	-
Avena strigosa	31	4	10	16	-	55	73	-
Balanites aegyptiaca	29	11	11	16	91	42	-	-
Bauhinia cheilantha	50	15	4	12	47	68	54	-
Bothriochloa insculpta	44	8	-	6	90	74	47	-
Bouteloua curtipendula	-	6	-	11	-	74	-	-
Brachiaria brizantha	47	20	2	8	22	73	62	-
Brachiaria decumbens	46	18	-	9	23	71	63	-
Brachiaria eruciformis	37	-	-	5	92	64	-	-
Brachiaria fasciculata	-	6	-	14	-	64	-	-
Brachiaria ruziziensis x decumbens	49	19	-	8	23	71	62	-
Brachypodium pinnatum	-	-	-	9	-	73	-	-
Bromus inermis	30	-	7	15	-	54	70	15
Calliandra calothyrsus	-	-	-	17	90	51	-	51
Capparis spinosa	30	-	14	23	-	41	-	-
Capparis tomentosa	27	10	10	18	92	45	-	-
Carum carvi	27	5	12	14	-	32	74	-
Cenchrus ciliaris	47	9	-	6	94	76	49	35
Centrosema pubescens	27	8	9	18	39	57	-	-
Chamaecrista rotundifolia	29	8	13	24	-	43	78	-
Chloris ciliata	-	4	-	13	-	70	-	-
Chloris pycnothrix	47	9	-	3	97	78	-	40
Chloris virgata	36	-	-	9	90	60	-	-
Chondrosum gracile	-	-	-	-	-	83	-	-
Chondrosum trifidum	-	6	-	11	-	74	-	-

Species	ADF	ADL	Ash	CP	DM	NDF	DMD	OMD
Cichorium intybus	28	4	14	10	-	33	72	-
Clitoria ternatea	43	6	4	15	29	60	65	-
Combretum molle	33	9	9	15	89	46	-	-
Cymbopogon caesius	40	-	-	7	90	62	-	-
Cynodon dactylon	45	-	-	17	25	78	55	-
Cynodon nlemfuensis	39	9	-	8	96	76	-	44
Dactylis glomerata	32	2	9	13	20	58	75	74
Dactyloctenium aegypticum	40	-	-	8	90	62	-	-
Dichrostachys cinerea	31	11	7	17	91	50	-	-
Digitaria abyssinica	38	5	10	11	90	73	-	-
Digitaria insularis	-	6	-	11	-	72	-	-
Diospyros abyssinica	35	11	19	14	90	48	-	-
Echinochloa crusgalli	36	-	-	12	-	62	-	-
Elymus gmelinii	-	-	5	15	-	-	-	-
Elymus hispidus	31	-	-	17	-	-	-	-
Elymus lanceolatus	32	-	-	16	-	-	-	-
Elymus nutans	-	-	6	14	-	-	-	-
Elymus sibiricus	34	-	-	14	-	-	61	34
Elymus smithii	-	-	-	18	-	-	-	-
Elymus trachycaulus	32	-	-	15	-	-	-	-
Enneapogon cenchroides	36	-	-	6	91	62	-	-
Enteropogon macrostachus	52	10	-	6	-	78	38	-
Eragrostis braunii	39	5	8	8	90	81	-	-
Eragrostis curvula	43	4	9	8	90	81	-	-
Eragrostis superba	33	-	-	8	90	63	-	-
Festuca arundinacea	29	2	8	14	21	60	71	-
Festuca arundinacea x Lolium multiflorum	-	-	-	8	-	58	-	74
Festuca pratensis	41	-	11	11	-	54	-	-
Festuca rubra	28	-	-	16	-	-	-	-
Gliricidia sepium	44	9	7	18	23	51	78	-
Grewia mollis	30	16	13	13	91	58	-	-
Heteropogon contortus	38	-	-	6	90	64	-	-
Hilaria belangeri	-	-	-	-	-	79	-	-
Holcus lanatus	33	-	-	11	-	54	-	-
Hordeum brevisubulatum	-	-	6	14	-	-	-	-
Hordeum vulgare	-	-	-	16	26	56	66	-
Hyparrhenia cymbaria	43	-	-	6	91	71	-	-
Hyparrhenia hirta	56	9	-	4	-	80	41	-
Kummerowia striata	-	-	-	17	-	-	-	73
Lablab purpureus	31	11	12	20	75	46	84	-
Lathyrus sativus	-	-	11	20	-	-	-	-
Lavandula angustifolia	41	-	9	10	-	50	-	-

Species	ADF	ADL	Ash	CP	DM	NDF	DMD	OMD
Leptochloa mucronata	-	6	-	12	-	70	-	-
Leucaena leucocephala	27	5	6	19	59	52	74	88
Lolium multiflorum	25	2	9	15	22	45	74	72
Lolium multiflorum × *Festuca pratensis*	-	-	11	12	-	-	-	-
Lolium perenne	31	2	9	18	18	49	74	81
Lolium perenne × *Festuca pratensis*	-	-	10	11	-	-	-	-
Lotus corniculatus	27	6	9	22	-	41	68	-
Macroptilium atropurpureum	33	9	17	28	-	42	74	-
Manihot pseudoglaziovii	34	10	7	10	22	43	79	-
Medicago sativa	30	7	10	21	17	42	71	67
Melilotus officinalis	27	5	11	20	-	33	70	-
Melinis repens	-	8	-	10	-	72	-	-
Mimosa caesalpiniifolia	55	16	4	12	44	68	45	-
Nassella clarazii	26	-	-	16	-	55	-	-
Noaea mucronata	50	-	9	10	-	59	-	-
Panicum coloratum	40	5	7	9	90	83	-	-
Panicum deustum	40	-	-	6	90	63	-	-
Panicum hallii	-	5	-	13	-	71	-	-
Panicum maximum	47	10	12	7	41	73	47	48
Panicum miliaceum	36	4	11	17	90	71	-	-
Panicum obtusum	-	6	-	14	-	65	-	-
Paspalum unispicatum	-	4	-	11	-	68	-	-
Pennisetum clandestinum	26	-	10	23	-	46	63	-
Pennisetum polystachion	40	-	2	7	19	78	-	-
Pennisetum purpureum	44	-	9	9	17	73	50	52
Phalaris aquatica	-	-	-	10	-	68	-	-
Phalaris arundinacea	31	-	-	-	-	60	-	-
Phleum pratense	31	3	7	15	23	52	88	-
Plantago lanceolata	34	8	10	12	-	40	64	-
Poa attenuata	35	-	-	13	-	-	57	18
Poa pratensis	29	-	-	15	-	56	-	-
Psophocarpus scandens	6	2	10	19	20	23	-	-
Sanguisorba minor	24	4	10	13	-	30	64	-
Sehima galpinii	37	-	-	5	91	65	-	-
Setaria grisebachii	-	6	-	14	-	72	-	-
Setaria incrassata	41	7	10	7	90	76	-	-
Setaria macrostachya	-	7	-	13	-	74	-	-
Stipa ichu	36	-	-	10	-	66	-	-
Stylosanthes guianensis	32	6	10	19	28	51	-	-
Terminalia brownii	36	14	11	14	90	50	-	-
Tragus berteronianus	43	-	-	5	90	78	-	-
Tridens eragrostoides	-	5	-	13	-	73	-	-
Tridens muticus	-	7	-	11	-	75	-	-

Species	ADF	ADL	Ash	CP	DM	NDF	DMD	OMD
Trifolium alexandrinum	-	-	-	18	-	-	-	71
Trifolium ambiguum	26	-	-	20	-	39	88	-
Trifolium pratense	28	5	12	19	16	41	76	-
Trifolium repens	24	-	-	25	29	38	88	-
Trifolium resupinatum	-	-	-	20	-	-	-	73
Triticosecale	-	-	10	8	-	-	-	-
Urochloa mosambicensis	43	-	-	6	91	66	-	-
Vicia pannonica	-	-	14	17	-	-	-	-
Vicia sativa	32	6	10	24	88	46	-	-
Vicia villosa	-	-	12	18	-	-	-	-
Vigna unguiculata	25	5	13	21	16	36	-	-
Ximenia americana	32	4	7	13	90	47	-	-
Ziziphus spina-christi	29	9	6	14	90	48	-	-

References

Ajayi FT, Babayemi OJ, Taiwo AA (2008) Effects of supplementation of Panicum maximum with four herbaceous forage legumes on performance, nutrient digestibility and nitrogen balance in West African dwarf goats. Anim Sci J 79:673–679. doi: 10.1111/j.1740-0929.2008.00579.x

Akgun I, Tosun M, Sengul S (2008) Comparison of agronomic characters of Festulolium, Festuca pratensis huds. and Lolium multiflorum lam. genotypes under high elevation conditions in Turkey. Bangladesh J Bot 37:1–6.

Albayrak S, Türk M (2013) Changes in the forage yield and quality of legume-grass mixtures throughout a vegetation period. Turkish J Agric For 37:139–147. doi: 10.3906/tar-1202-73

Al-Ghumaiz NS, Motawei MI (2011) Productivity, forage quality and presence of dehydrin genes in some introduced pasture grass cultivars growing under heat stress in central region of Saudi Arabia. Aust J Crop Sci 5:1001–1006.

Al-Masri MR (2013) Nutritive evaluation of some native range plants and their nutritional and anti-nutritional components. J Appl Anim Res 41:427–431. doi: 10.1080/09712119.2013.792733

Amole TA, Oduguwa BO, Onifade SO, et al (2015) Effect of planting patterns and age at harvest of two cultivars of Lablab purpureus in Andropogon gayanus on agronomic characteristic and quality of grass/legume mixtures. Pertanika J Trop Agric Sci 38:329–346.

Andrzejewska J, Contreras-Govea FE, Pastuszka A & Albrecht KA (2016) Performance of Kura clover compared to that of perennial forage legumes traditionally cultivated in central Europe, Acta Agric Scand B 66:6, 516-522, DOI: 10.1080/09064710.2016.1184306

Annese V, Cazzato E, Corleto A (2006) Quantitative and qualitative traits of natural ecotypes of perennial grasses (Dactylis glomerata L., Festuca arundinacea Schreb., Phalaris tuberosa L., Brachypodium rupestre (Host) R. et S.) collected in Southern Italy. Genet Resour Crop Evol 53:431–441. doi: 10.1007/s10722-004-1808-x

Beecher M, Hennessy D, Boland TM, et al (2015) The variation in morphology of perennial ryegrass cultivars throughout the grazing season and effects on organic matter digestibility. Grass Forage Sci 70:19–29. doi: 10.1111/gfs.12081

Bélanger G, Mcqueen RE (1997) Leaf and stem nutritive value of timothy cultivars differing in

maturity. Can J Plant Sci 772:237–249.

Berhane G, Eik L, Tolera A (2006) Chemical composition and in vitro gas production of vetch (Vicia sativa) and some browse and grass species in northern Ethiopia. African J. Range Forage Sci. 23:69–75.

Bryant RH, Gregorini P, Edwards GR (2012) Effects of N fertilisation, leaf appearance and time of day on N fractionation and chemical composition of Lolium perenne cultivars in spring. Anim Feed Sci Technol 173:210–219. doi: 10.1016/j.anifeedsci.2012.02.003

Callow MN, Lowe KF, Bowdler TM, et al (2003) Dry matter yield, forage quality and persistence of tall fescue (Festuca arundinacea) cultivars compared with perennial ryegrass (Lolium perenne) in a subtropical environment. Aust J Exp Agric 43:1093–1099. doi: 10.1071/EA02001

Catanese F, Distel RA, Arzadún M (2009) Preferences of lambs offered Italian ryegrass (Lolium multiflorum L.) and barley (Hordeum vulgare L.) herbage as choices. Grass Forage Sci 64:304–309. doi: 10.1111/j.1365-2494.2009.00698.x

Cherney DJR, Cherney JH (1997) Grass forage quality and digestion kinetics as influenced by nitrogen fertilization and maturity. J Appl Anim Res 11:105–120. doi: 10.1080/09712119.1997.9706170

Conaghan P, O'Kiely P, Howard H, et al (2008) Evaluation of Lolium perenne L. cv. AberDart and AberDove for silage production. Irish J Agric Food Res 47:119–134.

Čop J, Lavrenčič A, Košmelj K (2009) Morphological development and nutritive value of herbage in five temperate grass species during primary growth: Analysis of time dynamics. Grass Forage Sci 64:122–131. doi: 10.1111/j.1365-2494.2008.00676.x

Distel RA, Didoné NG, Moretto AS (2005) Variations in chemical composition associated with tissue aging in palatable and unpalatable grasses native to central Argentina. J Arid Environ 62:351–357. doi: 10.1016/j.jaridenv.2004.12.001

Dong SK, Long RJ, Hu ZZ, et al (2003) Productivity and nutritive value of some cultivated perennial grasses and mixtures in the alpine region of the Tibetan Plateau. Grass Forage Sci 58:302–308. doi: 10.1046/j.1365-2494.2003.00382.x

dos Santos M, Junior J, Silva M, et al (2003) Productivity and chemical composition of tropical grasses in the forest zone of Pernambuco. Rev Bras Zootec 32:821–827.

Drobná J, Jančovič J (2006) Estimation of red clover (Trifolium pratense L.) forage quality parameters depending on the variety, cut and growing year. Plant, Soil Environ 52:468–475.

Elgersma A, Søegaard K, Jensen SK (2014) Herbage dry-matter production and forage quality of three legumes and four non-leguminous forbs grown in single-species stands. Grass Forage Sci 69:705–716. doi: 10.1111/gfs.12104

Fraser MD, Speijers MHM, Theobald VJ, et al (2004) Production performance and meat quality of grazing lambs finished on red clover, lucerne or perennial ryegrass swards. Grass Forage Sci 59:345–356. doi: 10.1111/j.1365-2494.2004.00436.x

Gierus M, Kleen J, Loges R, Taube F (2012) Forage legume species determine the nutritional quality of binary mixtures with perennial ryegrass in the first production year. Anim Feed Sci Technol 172:150–161. doi: 10.1016/j.anifeedsci.2011.12.026

Griggs TC, MacAdam JW, Mayland HF, Burns JC (2007) Temporal and vertical distribution of nonstructural carbohydrate, fiber, protein, and digestibility levels in orchardgrass swards. Agron J 99:755–763. doi: 10.2134/agronj2006.0036

Haferkamp M, Grings E (2002) Quality and persistence of forages in the Northern Great Plains. J Range Manag 55:482–487.

Hirata M, Islam M, Harada E, et al (2008) Sward structure and herbage quality, production and utilisation of adjacent monocultures of centipede grass and bahia grass grazed by cattle. Trop Grasslands 48:202–213.

Homolka P, Koukolová V, Podsedníček M, Hlaváčková A (2012) Nutritive value of red clover and lucerne forages for ruminants estimated by in vitro and in vivo digestibility methods. Czech J Anim Sci 57:454–468.

Kambashi B, Picron P, Boudry C, et al (2014) Nutritive value of tropical forage plants fed to pigs in the Western provinces of the Democratic Republic of the Congo. Anim Feed Sci Technol 191:47–56. doi: 10.1016/j.anifeedsci.2014.01.012

Karadag Y, Buyukburc U (2004) Forage qualities, forage yields and seed yields of some legume-triticale mixtures under rainfed conditions. Acta Agric Scand Sect B Soil Plant Sci 54:140–148. doi: 10.1080/09064710310015481

Keating T, O'Kiely P (2000) Comparison of old permanent grassland, Lolium perenne and Lolium multiflorum swards grown for silage 4. Effects of varying harvesting date. Irish J Agric Food Res 39:55–71.

King C, McEniry J, Richardson M, O'Kiely P (2012) Yield and chemical composition of five common grassland species in response to nitrogen fertiliser application and phenological growth stage. Acta Agric Scand Sect B-Soil Plant Sci 62:644–658. doi: 10.1080/09064710.2012.687055

Kobayashi H, Takahashi Y, Matsumoto K, Nishiguchi Y (2008) Changes in nutritive value of italian ryegrass (Lolium multiflorum Lam.) during overwintering period. Plant Prod Sci 11:228–231.

Lee TD, Tjoelker MG, Ellsworth DS, Reich PB (2001) Leaf gas exchange responses of 13 prairie grassland species to elevated CO2 and increased nitrogen supply. New Phytol 150:405–418. doi: 10.1046/j.1469-8137.2001.00095.x

Maasdorp B V., Muchenje V, Titterton M (1999) Palatability and effect on dairy cow milk yield of dried fodder from the forage trees Acacia boliviana, Calliandra calothyrsus and Leucaena leucocephala. Anim Feed Sci Technol 77:49–59. doi: 10.1016/S0377-8401(98)00232-6

McCartney DH, Lardner HA, Stevenson FC (2008) Economics of backgrounding calves on Italian ryegrass (Lolium multiflorum) pastures in the Aspen Parkland. Can J Anim Sci 88:19–28. doi: 10.4141/CJAS07064

Mceniry J, King C, O'Kiely P (2014) Silage fermentation characteristics of three common grassland species in response to advancing stage of maturity and additive application. Grass Forage Sci 69:393–404. doi: 10.1111/gfs.12038

Mtengeti EJ, Phiri ECJH, Urio NA, et al (2008) Forage availability and its quality in the dry season on smallholder dairy farms in Tanzania. Acta Agric Scand A Anim Sci 58:196–204. doi: 10.1080/09064700802492362

Nashiki M, Narita H, Higashiyama Y (2005) Herbage mass, nutritive value and palatability of five grass weeds for cattle in the northern Tohoku region in Japan. Weed Biol Manag 5:110–117. doi: 10.1111/j.1445-6664.2005.00171.x

Ramirez RG (2007) In situ Digestibility of Neutral Detergent Fiber of Introduced Cenchrus ciliaris and Six Native Mexican Grasses Consumed by Small Ruminants. J Appl Anim Res 31:53–57. doi: 10.1080/09712119.2007.9706629

Safari J, Mushi DE, Kifaro GC, et al (2011) Seasonal variation in chemical composition of native forages, grazing behaviour and some blood metabolites of Small East African goats in a semi-arid area of Tanzania. Anim Feed Sci Technol 164:62–70. doi: 10.1016/j.anifeedsci.2010.12.004

Sahin E, Tosun M, Haliloglu K (2012) Some agricultural and quality properties of ulubag ecotype lines of wild orchardgrass (Dactylis glomerata L.). Turkish J F Crop 17:191–197.

Santos D de C, Guimarães Júnior R, Vilela L, et al (2016) Forage dry mass accumulation and structural characteristics of Piatã grass in silvopastoral systems in the Brazilian savannah. Agric Ecosyst Environ 233:16–24. doi: 10.1016/j.agee.2016.08.026

Skladanka J, Adam V, Ryant P, et al (2010) Can Festulolium, Dactylis glomerata and Arrhenatherum elatius be used for extension of the autumn grazing season in central Europe? Plant, Soil Environ 56:488–498.

Smit HJ, Tas BM, Taweel HZ, et al (2005) Effects of perennial ryegrass (Lolium perenne L.) cultivars on herbage production, nutritiional quality and herbage intake of grazing dairy cows. Grass Forage Sci 60:297–309.

Suleiman A, Okine EK, Goonewardene LA, et al (1999) Yield and feeding of prairie grasses in east-central Alberta. J Range Manag 52:75–82.

Surmen M, Yavuz T, Albayrak S, Cankaya N (2013) Forage yield and quality of perennial ryegrass (Lolium perenne L.) lines in the black sea coastal area of Turkey. Turkish J F Crop 18:40–45.

Taffarel LE, Mesquita EE, Castagnara DD, et al (2016) Tifton 85 grass responses to different nitrogen levels and cutting intervals. Semin Agrar 37:2067–2084. doi: 10.5433/1679-0359.2016v37n4p2067

Tas BM, Taweel HZ, Smit HJ, et al (2005) Effects of perennial ryegrass cultivars on intake, digestibility, and milk yield in dairy cows. J Dairy Sci 88:3240–3248. doi: 10.3168/jds.S0022-0302(05)73007-1

Tefera S, Mlambo V, Dlamini BJ, et al (2009) Chemical composition and in vitro ruminal fermentation of selected grasses in the semiarid savannas of Swaziland. African J Range Forage Sci 26:9–17. doi: 10.2989/ajrfs.2009.26.1.2.697

Ulyatt MJ, Lassey KR, Shelton ID, Walker CF (2002) Methane emission from dairy cows and wether sheep fed subtropical grass-dominant pastures in midsummer in New Zealand. New Zeal J Agric Res 45:227–234. doi: 10.1080/00288233.2002.9513513

Weller RF, Cooper A (2001) Seasonal changes in the crude protein concentration of mixed swards of white clover/perennial ryegrass grown without fertilizer N in an organic farming system in the United Kingdom. Grass Forage Sci 56:92–95. doi: 10.1046/j.1365-2494.2001.00248.x

Yayneshet T, Eik LO, Moe SR (2009) Seasonal variations in the chemical composition and dry matter degradability of exclosure forages in the semi-arid region of northern Ethiopia. Anim Feed Sci Technol 148:12–33. doi: 10.1016/j.anifeedsci.2008.02.003

Zemenchik RA, Albrecht KA, Shaver RD (2002) Improved nutritive value of kura clover- and birdsfoot trefoil-grass mixtures compared with grass monocultures. Agron J 94:1131–1138. doi: 10.2134/agronj2002.1131

Zhao Y, Ma M, Li X (2012) Nutritional value and amino acid content of four grasses in Eastern Inner Mongolia. J Anim Vet Adv 11:3928–3936.
